# Nintedanib-induced lichenoid drug eruption: a case report

**DOI:** 10.1093/skinhd/vzag051

**Published:** 2026-05-07

**Authors:** Mahesh Mathur, Sumit Paudel, Sambidha Karki, Nabita Bhattarai, Sharad Shrestha, Sandhya Regmi

**Affiliations:** Department of Dermatology, College of Medical Sciences Teaching Hospital, Bharatpur, Nepal; Department of Dermatology, College of Medical Sciences Teaching Hospital, Bharatpur, Nepal; Department of Dermatology, College of Medical Sciences Teaching Hospital, Bharatpur, Nepal; Department of Dermatology, College of Medical Sciences Teaching Hospital, Bharatpur, Nepal; Department of Dermatology, College of Medical Sciences Teaching Hospital, Bharatpur, Nepal; Department of Dermatology, College of Medical Sciences Teaching Hospital, Bharatpur, Nepal

## Abstract

Lichenoid drug eruption is a cutaneous adverse reaction that resembles lichen planus but is distinguished by marked polymorphism, including lichenoid, psoriasiform and eczematous lesions. It is a type IV hypersensitivity reaction that has been ­associated with the use of numerous medications, including angiotensin-converting enzyme inhibitors, thiazide diuretics, antimalarials and beta blockers. Nintedanib works as an intracellular inhibitor of tyrosine kinases, targeting numerous receptors, including vascular endothelial growth factor, fibroblast growth factor and platelet-derived growth factor. We report a case of lichenoid drug eruption secondary to nintedanib. This association has not previously been described in the literature.

## Teaser

Lichenoid drug eruption is a cutaneous adverse reaction that resembles lichen planus but is distinguished by marked polymorphism, including lichenoid, psoriasiform and eczematous lesions. Nintedanib is a tyrosine kinase inhibitor that has antifibrotic, anti-inflammatory and angiogenesis attenuation effects. We hereby report a case of lichenoid drug eruption secondary to nintedanib that has not been described in the literature so far.

What is already known about this topic?

- Lichenoid drug eruption is a type IV hypersensitivity reaction that has been associated with the use of numerous medications, including angiotensin-converting enzyme inhibitors, thiazide diuretics, antimalarials and beta blockers.

What does this study add?

- We report a case of lichenoid drug eruption secondary to nintedanib.
- This association has not previously been described in the literature.

Lichenoid drug eruption is an uncommon cutaneous adverse drug reaction characterized by symmetrical lichenoid, eczematous or psoriasiform lesions mainly on photodistributed regions on the body. It is a type IV hypersensitivity reaction that has been associated with the use of numerous medications, including ­angiotensin-converting enzyme inhibitors, thiazide diuretics, antimalarials and beta blockers.^1,2^

Nintedanib is a tyrosine kinase inhibitor that has antifibrotic, anti-inflammatory and angiogenesis attenuation effects. It is used for treating idiopathic pulmonary fibrosis, systemic sclerosis-associated interstitial lung disease, the progressive phenotype of interstitial lung disease and non-small cell lung cancer.^3^ Here we report a case of lichenoid drug eruption secondary to nintedanib, an association that has not previously been described in the literature.

## Case report

**Figure 1:**
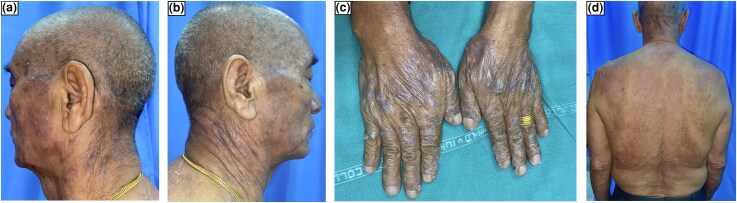
Multiple violaceous papules and plaques over (a, b) the neck and (c) the dorsum of the hands bilaterally, and (d) psoriasiform lesions over the back.

A 66-year-old man with interstitial lung disease presented with itchy violaceous papules and plaques on the neck and dorsal aspect of the bilateral hands, and psoriasiform lesions on the back for 3 months (Figure 1). He had been diagnosed with pulmonary tuberculosis 2 years earlier and completed a 6-month course of antitubercular therapy. Subsequently, he developed interstitial lung disease and was initially treated with inhaled formoterol fumarate and budesonide, with minimal clinical benefit. Four months prior to presentation, oral nintedanib monotherapy at a dose of 150 mg twice daily was initiated. One month after starting nintedanib, he developed the cutaneous lesions, for which topical steroids were prescribed, resulting in minimal improvement. Examination of the mucosae did not reveal any lesions.

**Figure 2:**
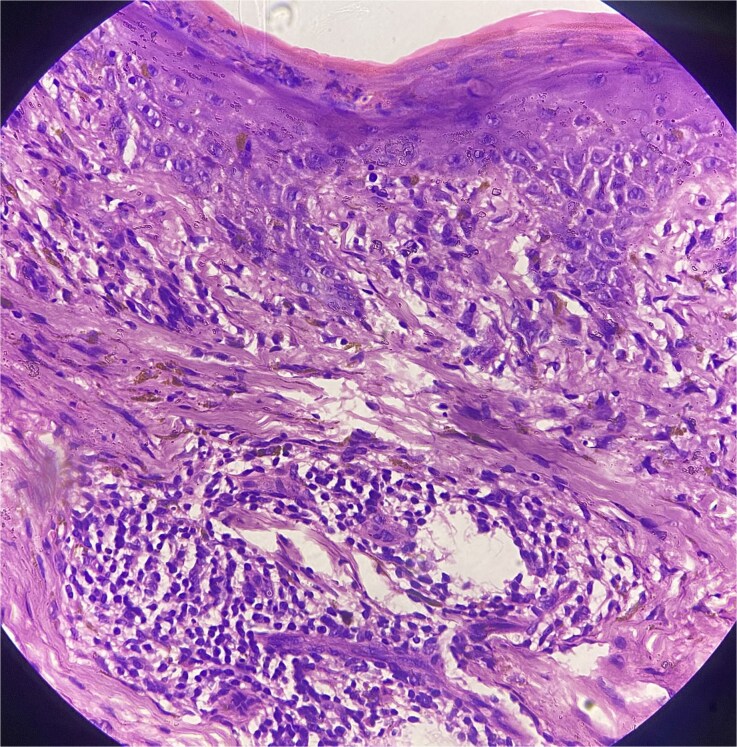
Haematoxylin and eosin staining (original magnification ×40) showed parakeratosis, loss of granular layer, interface dermatitis, melanin incontinence and rare eosinophils in the upper dermis.

Routine blood investigations were within normal limits. Skin biopsy showed parakeratosis, loss of the granular layer, lymphocytic infiltrate at the interface, melanin incontinence and rare eosinophils in the upper dermis (Figure 2). The Naranjo adverse drug reaction probability scale value was calculated to be 9, which indicates a definitive adverse drug reaction to nintedanib.^4^ Based on the clinical and histological findings, a diagnosis of lichenoid drug eruption secondary to nintedanib was made.

Nintedanib was stopped after discussion with the treating physician, and the patient was started on oral prednisolone 40 mg daily (approximately 0.5 mg kg^−1^ daily). Over the next 10 days, his pruritus subsided, and the skin lesions began to improve, leaving postinflammatory hyperpigmentation. The oral steroid was then gradually tapered over 3 weeks, and there was no recurrence of lesions during follow-up.

## Discussion

Nintedanib works as an intracellular inhibitor of tyrosine kinases, targeting numerous receptors, including vascular endothelial growth factor, fibroblast growth factor and platelet-derived growth factor.^3,5^ The most common adverse effects of nintedanib are gastrointestinal events, mainly diarrhoea, vomiting and abdominal pain.^3^ Adverse cutaneous side effects from nintedanib are rarely reported and include pruritus, dermal ulcer, acneiform eruption, bullous pemphigoid and photosensitive dermatitis.^3,5–7^

Although the pathogenesis of lichenoid drug eruption is not clearly understood, it has been suggested that cross-reactivity between the drug and keratinocytes, both acting as antigens, leads to T-cell-mediated autoimmune damage of basal keratinocytes.^1,3^ The blockage of multiple tyrosine kinase receptors altering the antigenic properties of the epidermal basement membrane may be the triggering factor for nintedanib-induced lichenoid drug eruption.^5^ The latency period between ingestion of the offending drug and onset of lichenoid drug eruption has been reported to range from 15 days to 6 months. Our patient developed lesions after 1 month of intake of the offending medication.^1^

Lichenoid drug eruption is a cutaneous adverse reaction that resembles lichen planus but is distinguished by marked polymorphism, including lichenoid, psoriasiform and eczematous lesions. Unlike lichen planus, it typically lacks Wickham striae, tends to appear in photodistributed areas and rarely affects mucosal surfaces.^1,2^ Histopathologically, the presence of varying degrees of eosinophilic infiltrates, focal parakeratosis and focal interruption of the granular layer and deep perivascular infiltrate helps to differentiate lichenoid drug eruption from lichen planus, as seen in our case.^1^

Treatment includes discontinuation of the offending medication, which often leads to resolution of lesions within weeks to months. If the drug cannot be discontinued, or the patient has prolonged or extensive disease, therapies for idiopathic lichen planus can be used, including topical, intralesional and systemic corticosteroids, topical calcineurin inhibitors, narrowband ultraviolet B phototherapy, and systemic retinoids.^1,2^

In conclusion, the recognition of lichenoid drug eruption is important, as withdrawal of the culprit drug often leads to rapid resolution of symptoms with no recurrence. This case is reported to highlight the possibility of lichenoid drug eruption as a rare but potential adverse effect of nintedanib, emphasizing the need for clinical vigilance.

## Data Availability

The data underlying this article will be shared on reasonable request to the corresponding author.
